# Endothelialization of an ePTFE vessel prosthesis modified with an antithrombogenic fibrin/heparin coating enriched with bound growth factors†

**DOI:** 10.1039/d1ra00053e

**Published:** 2021-02-03

**Authors:** Johanka Táborská, Zuzana Riedelová, Eduard Brynda, Pavel Májek, Tomáš Riedel

**Affiliations:** Institute of Macromolecular Chemistry, Czech Academy of Sciences Heyrovského Náměstí 2 162 06 Prague 6 Czech Republic riedel@imc.cas.cz; Institute of Hematology and Blood Transfusion U Nemocnice 1 128 00 Prague 2 Czech Republic

## Abstract

Early and late thrombosis remain the most frequent reasons for the failure of synthetic cardiovascular grafts. Long-term hemocompatibility of implanted synthetic grafts can be achieved if a natural living endothelium is formed over its blood-contacting surface. Here we present a modification of a standard expanded polytetrafluorethylene (ePTFE) vessel prosthesis by a controlled preparation of a fibrin mesh enriched with covalently bound heparin and noncovalently bound vascular endothelial growth factor (VEGF) and fibroblast growth factor (FGF). Compared to a bare prosthesis, the coated prosthesis showed excellent antithrombogenic properties after contact with heparinized fresh human blood. Human umbilical vein endothelial cells seeded on the inner surface of the coated prosthesis formed a confluent layer in 5 days, whereas only small colonies of cells were scattered on the bare prosthesis. Viability of the cells was promoted mainly by FGF immobilized on the coating. These findings suggest that the coating may prevent acute thrombus formation and support the self-endothelialization of an implanted ePTFE vascular graft *in vivo*.

## Abstract 2

A fibrin/heparin coating enriched with bound growth factors was developed and applied on an ePTFE vessel prosthesis. The coating exhibited excellent antithrombogenic properties and supported endothelialization of the ePTFE vessel.

## Graphical abstract

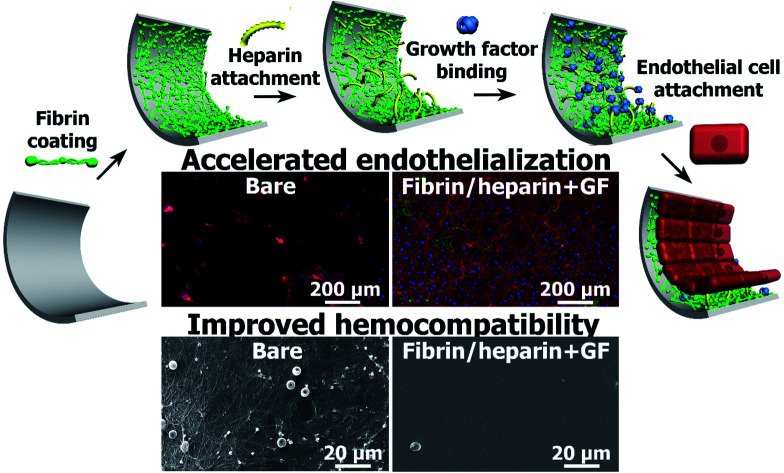

## Introduction

Cardiovascular diseases are the leading cause of death worldwide. Blood flow restriction due to the narrowing of the vessel lumen or vessel blockage leads to ischemia, which results in life-threatening conditions. To replace or bypass a damaged vessel, vascular grafts (VGs) are used. Currently, the best conduits for vascular grafting are autologous arteries or veins, typically the saphenous veins, internal mammary arteries, or radial arteries.^1,2^ Nevertheless, they have several drawbacks: limited availability; donor site morbidity and patient burden due to the requirement for additional surgery. As a result, synthetic polymer VGs made from expanded polytetrafluoroethylene (ePTFE), polyethylene terephthalate (PET, Dacron), and polyurethane (PU) are routinely used in current clinical practice as an alternative to autologous vessels.^3^ However, all synthetic grafts suffer from occasional acute, subacute, or late thrombotic complications. The risk of rapid thrombotic occlusion limits the application of synthetic VGs of less than 4 mm in diameter.^4,5^ The formation of a healthy natural endothelium over the luminal surface of an implanted VG seems to be a key step in reaching long-term hemocompatibility of synthetic VGs.^6,7^ The inability to form an endothelial lining leads to high failure rates in small and medium-sized synthetic VGs.^4,5^

The endothelialization of the implanted synthetic VG's luminal surface includes the migration of vascular endothelial cells (ECs) from the anastomotic area (transanastomotic), ingrowth of ECs and microvessels from the perigraft tissue if the graft contains sufficiently large pores (transmural), and the adherence of circulating endothelial progenitor cells (EPCs) from peripheral blood (blood-borne). However, none of these processes are capable of spontaneously developing a neo-intima on VGs in humans, except for sporadically observed small islands of ECs.^8^*In vitro* pre-seeding of VGs with autologous ECs may help the endothelialization after surgery. Nevertheless, the surgical harvesting of ECs from the patient, the *in vitro* ECs expansion and cultivation on the graft are time consuming and burdened with the risk of infection.^8,9^

On the other hand, *in situ* endothelialization is a promising method based on a graft modification that uses a coating which accelerates the self-endothelialization after the graft implantation while suppressing thrombosis until a functional neointima is formed.^9,10^ A design of the graft modification might be inspired by natural wound healing processes that lead to the regeneration of the endothelium after vascular injury. A blood clot, which is composed of a crosslinked fibrin mesh containing activated and aggregated platelets, erythrocytes, and leukocytes, is formed at sites of endothelium damage to stop bleeding and later serves as a temporary scaffold for ECs migrating from the neighbouring intact endothelium and for EPCs captured from peripheral blood.^11–13^ In addition, the repair of injured blood vessels is stimulated by various growth factors and cytokines released from activated platelets and leukocytes trapped in the fibrin mesh. The endothelium regeneration is particularly accelerated by vascular endothelial growth factor (VEGF) and fibroblast growth factors (FGF) accumulated in the blood clot by the attachment to specific binding sites in the fibrin structure.^14,15^ VEGF supports also adhesion, proliferation, differentiation and the maturation of various cells including EPCs and FGF supports the proliferation of smooth muscle cells and extracellular matrix remodelling in the injured vessel wall.^16–19^

Artificial fibrin meshes have been widely used for the modification of VGs as biodegradable substrates supporting adhesion and migration of ECs and EPCs.^20–24^ A significant improvement in the patency of ePTFE vascular grafts was observed when they were coated with a fibrin glue and endothelialized *in vitro* by the seeding of autologous endothelial cells before implantation.^25^ Fibrin glue is prepared by mixing a highly concentrated fibrinogen (Fbg) solution containing factor XIII with a thrombin solution immediately before use. In order to promote the *in situ* endothelialization, synthetic polymer VGs were modified with fibrin containing immobilized FGF and/or VEGF. A significant increase in luminal ECs proliferation and transmural capillary ingrowth was observed in ePTFE grafts modified by fibrin glue containing FGF factors in canine aortic models.^26^ On the other hand, an ePTFE graft modified with fibrin and genetically engineered recombinant VEGF showed effects deleterious to graft healing in a pig carotid artery interposition model, by increasing narrowing at the proximal anastomosis and neointimal hyperplasia, and by increased platelet deposition and thrombus formation in extracorporeal arteriovenous shunt perfusion.^27^

To reduce thrombogenicity in synthetic polymer VGs, the grafts are usually modified with immobilized anticoagulant heparin. ePTFE vascular grafts with covalently bound heparin significantly reduced platelet adhesion and thrombus formation both *in vitro* and *in vivo*.^28^ We have previously shown that a covalent binding of periodate activated heparin to a fibrin mesh prepared on PVC sheets prevented the formation of a blood clot on the sheets exposed to human blood in a Chandler loop model.^29^

The present work aims to develop a surface modification that might accelerate the *in vitro* and *in situ* endothelialization of cardiovascular grafts while suppressing acute thrombosis. The modification of standard ePTFE grafts with a fibrin mesh decorated by the covalently bound heparin and noncovalently bound FGF and VEGF presented in this work fulfils both requirements. The modification includes the controlled formation of a fibrin mesh (Fb) at a substrate surface using adapted techniques that we developed earlier^29–31^ followed by the covalent binding of chemically activated heparin to Fb and finally by the attachment of VEGF and FGF *via* their specific noncovalent binding to fibrin^14,32^ and heparin.^33,34^ The controlled formation of the fibrin mesh consists of three successive processes: (1) fibrinogen adsorption on the substrate, (2) thrombin attachment to the adsorbed fibrinogen monolayer, and (3) fibrin formation by the catalytic action of the bound thrombin on the fibrinogen in an ambient solution. Unlike the conventional mixing of fibrinogen solution with thrombin (*e.g.*, fibrin glue), which leads to the spontaneous formation of bulky fibrin meshes and glues, the independent steps taken in our procedure make it possible to reproducibly control the structure and the thickness of the fibrin mesh formed at the surface of a substrate (including a surface of inner pores). The covalent attachment of heparin to the fibrin suppressed the blood clot formation on the prosthesis surface exposed to human blood. The further noncovalent binding of FGF and VEGF to the fibrin mesh did not affect the antithrombogenic properties of the prosthesis and accelerated the *in vitro* formation of a confluent monolayer from human umbilical vein endothelial cells (HUVECs) seeded on the luminal prosthesis surface.

## Materials and methods

### Materials and reagents

Fibrinogen (Fbg) and thrombin (Thr) from human plasma, heparin sodium salt from porcine intestinal mucosa, plasmin, Phalloidin – Atto 594, TWEEN® 20, Triton™ X-100, paraformaldehyde, bovine serum albumin (BSA), NaIO_4_, NaOH, NaCl, CaCl_2_, KCl, Na_2_HPO_4_·12H_2_O, KH_2_PO_4_, citric acid monohydrate, sodium citrate – tribasic dehydrate, Trizma© base, agarose, and glutaraldehyde were purchased from Sigma-Aldrich. Antithrombin III, and COATEST© Heparin KIT were purchased from Chromogenix. Human fibroblast growth factor-basic 154aa (FGF) and human vascular endothelial growth factor 165 (VEGF) were purchased from GenScript USA, Inc. Cell Counting Kit-8 (CCK-8 assay) was purchased from Dojindo. Blotting-Grade Blocker was purchased from Bio-Rad. Hoechst 33342, the VEGF Human ELISA Kit (KHG0111), the FGF-2 Human ELISA Kit (KHG0021), the Micro BCATM Protein Assay Kit, Fibrinogen Polyclonal Antibody (PA1-9526), and Goat anti-Chicken IgY Alexa Fluor 488 (A11039) were purchased from Thermo Fisher Scientific. Amicon Ultra-4 centrifugal filter devices were purchased from Merck Millipore. 24- and 96- glass bottom well plates were purchased from BIO-PORT Europe. Phosphate-buffered saline pH 7.4 (PBS), Tris–HCl buffer 0.05 M with 2 mM CaCl_2_ pH 7.4 (TB), and 0.01 M citrate buffer pH 4.0 (CB) were filtered through a Millipore 0.22 μm filter. Human umbilical vein endothelial cells (HUVECs) were purchased from Promocell and maintained in endothelial cell growth medium (EGM-2, Lonza). Expanded polytetrafluorethylene vascular prostheses (ePTFE vessel) were kindly provided by B. Braun Medical.

### Fibrin (Fb) and fibrin with covalently bound heparin (Fb/Hep) coatings

#### Preparation of the fibrin coating (Fb)

A fibrin mesh was grown on the surface of wells in 24- or 96- glass bottom well plates and on flat samples (2 cm^2^) cut from an ePTFE vessel prothesis by adapting our previously developed technique.^30^ The technique is based on the controlled formation of a fibrin mesh from a substrate surface by catalytical activity of surface-bound thrombin.

#### Coating of the glass with Fb

The procedure consists of successive exchanging solutions in a glass bottom well (0.3 and 0.1 ml per well in 24- and 96- well plate, respectively) as follows: (1) Fbg 5 μg ml^−1^ in TB for 2 h (Fbg adsorption); (2) thrombin 2 U ml^−1^ in TB for 15 minutes (thrombin binding); and (3) ATIII 0.5 U ml^−1^ and Fbg 200 μg ml^−1^ in TB for 1 h (formation of Fb) at room temperature (RT). The surface was washed with TB after every step.

#### Modification of ePTFE samples

Flat ePTFE samples were fixed to a 24-well plate by CellCrown™ inserts and sterilized by 70% ethanol for 1 h. Consequently, samples were immersed in PBS and further treated with the same sequence of procedures as described above with prolonged incubation times: (1) Fbg was adsorbed overnight; (2) thrombin binding was extended to 30 min; and (3) fibrin formation was prolonged to 2 h.

#### Binding of heparin to Fb

Heparin was covalently bound to Fb by the reaction of primary amine groups on Fb with aldehyde groups on heparin prepared by periodate oxidation.^29^ First heparin (14 mg ml^−1^) was reacted with NaIO_4_ (2.4 mg ml^−1^) in PBS for 90 minutes at RT in the dark. Following this the solution of heparin aldehyde was desalted three times by repeated centrifugation at 7200 × *g* for 30 min using an Amicon Ultra-4 centrifugal filter device. Finally, the activated heparin solution was diluted in CB to the initial reaction volume and immediately added to the fibrin network. After 2 h, NaBH_3_CN (3.15 mg ml^−1^ in 0.1 M NaOH, final concentration) was added to the heparin aldehyde solution, and this was kept overnight at 4 °C. On the following day the resulting fibrin coating with covalently attached heparin (Fb/Hep) was washed with PBS and stored at 4 °C.

### Characterization of coatings

#### Fibrin concentration and heparin activity

The amount of fibrin in the Fb coating on the glass or ePTFE vessels was measured by a Micro BCA™ Protein Assay Kit according to the Thermo Scientific manual, and the mass of fibrin per cm^2^ was calculated. The activity of heparin bound in the Fb/Hep coating on the glass or ePTFE vessels was measured by a COATEST Heparin Kit.

#### Scanning electron microscopy (SEM)

Fibrin coatings prepared on the glass, ePTFE vessels prothesis, and samples after blood contact were dehydrated by successive incubation with aqueous solutions containing gradually increasing ethanol concentrations. The dehydrated surfaces were platinum sputtered and observed by SEM using a VEGA Plus TS 5135 microscope (Tescan, Brno, Czech Republic).

#### Atomic force microscopy (AFM)

Fb coatings prepared on glass were dried by successive ethanol dehydration and observed with an atomic force microscope (Dimension ICON, Bruker, Germany). A longitudinal silicon cantilever OTESPA-R3 designed for tapping mode in air (160 μm, spring constant 26 N m^−1^, tip radius 7 nm) was used for the AFM measurements. The images were evaluated using Gwyddion 2.56 data analysis software (www.gwyddion.net). For each sample, a series of images were collected demonstrating the uniformity of the fibrin.

#### Immunofluorescence staining of fibrin for confocal microscopy

Fibrin coatings on glass and ePTFE vessel samples were subjected to immunofluorescence staining by incubation with a fibrinogen antibody (1 : 2000) for 2 h in a 1% solution of Blotting-Grade Blocker (non-fat dry milk) in PBS-T (PBS + 0.02% Tween 20) and subsequent incubation with Goat anti-Chicken IgY Alexa Fluor 488 (1 : 4000) in PBS-T containing 1% Blotting-Grade Blocker for 1 h at RT. To visualize fibrin inside the ePTFE vessel wall, a flat sample of the Fb coated ePTFE vessel was fixed in 4% agarose gel and slices (80 μm thickness) were cut from the sample by a Vibratome Leica VT1200. The cut samples were subjected to immunofluorescence staining by incubation with a fibrinogen antibody (1 : 2000) for 72 h in a 1% solution of Blotting-Grade Blocker in PBS-T at 4 °C followed by incubation with Goat anti-Chicken IgY Alexa Fluor 488 antibody (1 : 4000) in PBS-T containing 1% Blotting-Grade Blocker for 2 h. Stained samples were observed using an Olympus IX83 confocal microscope.

### GFs binding to and release from Fb/Hep coatings

#### Binding of growth factors (GFs) to Fb/Hep coatings

The Fb/Hep coatings with bound FGF or VEGF (Fb/Hep + FGF, Fb/Hep + VEGF) were prepared by incubating the Fb/Hep coated 24-glass bottom well plates with solutions of VEGF or FGF at concentrations of 10 ng, 100 ng, 1 μg, and 10 μg per 1 ml in PBS. Fb/Hep containing both FGF and VEGF (Fb/Hep + FGF_100_ + VEGF_100_) was prepared by incubating the Fb/Hep coating with a 100 ng ml^−1^ co-solution of both FGF and VEGF in PBS. After 2 h incubation at RT the unbound GFs were removed by repeated rinsing with PBS (5×). To evaluate the amount of FGF and VEGF, the coating was degraded by plasmin (0.01 U ml^−1^ PBS) for 2 h at RT. The 2 h incubation with plasmin was sufficient to dissolve the fibrin mesh and to release the attached GFs. The amounts of the GFs in the obtained solutions were determined by ELISA kits following the producer's manual.

#### Release of FGF and VEGF from Fb/Hep coatings

The release of GFs was analysed for Fb/Hep samples with bound FGF or VEGF that were prepared by incubating Fb/Hep with a solution of respective GFs at the concentration of 100 ng ml^−1^ (Fb/Hep + FGF_100_, Fb/Hep + VEGF_100_). After a 2 h incubation followed by a brief washing with PBS, the wells were filled with 0.3 ml of fresh PBS. A sample of the PBS solution containing the released GFs was collected after 2 h, 4 h, 6 h, 24 h, 48 h, 72 h, 168 h and 336 h and replaced with a fresh PBS. The experiment was performed at 4 °C and the collected samples were stored at −80 °C until analysis. In a simultaneous experiment, the coatings (Fb/Hep + VEGF_100_ and Fb/Hep + FGF_100_) were briefly washed with PBS and incubated in PBS for 0, 7 or 14 days and the amounts of GFs remaining in the coating were determined by ELISA after lysis by plasmin as described above.

### Hemocompatibility of coatings

#### Plasma recalcification test

Fb and Fb/Hep coatings prepared in 24–well glass bottom plates were incubated with citrated human blood plasma freshly recalcified with CaCl_2_ (0.02 M final concentration) and light transmission through the coated glass bottom and the layer of the recalcified plasma was measured at 350 nm by a microplate reader (Epoch, Biotek) every 20 seconds for 4 h. The transmission decreased due to the turbidity of the plasma clot formed on the coated surface. The plasma recalcification time (PRT) was defined for our experiments as the time from adding freshly recalcified plasma into a well to half-minimum in the S-shaped transmission *versus* time curve.

#### Contact with heparinized blood

The hemocompatibility of the fibrin coated samples (on glass and ePTFE) after contact with freshly drawn human blood was evaluated using scanning electron microscopy. The blood was collected by venepuncture from healthy volunteers (*n* = 3) and anticoagulated with heparin (final concentration 1 IU ml^−1^). The coated glass and ePTFE samples were incubated with freshly drawn blood for 60 min under mild shaking at 37 °C. Subsequently, the samples were thoroughly washed with PBS, crosslinked with 0.5% glutaraldehyde for 2 h at RT, washed again and prepared for SEM as described above. All samples were obtained in accordance with the Ethics Committee Regulations of the Institute of Haematology and Blood Transfusion, Prague, and with informed consent.

### Viability and growth of HUVECs seeded on coated substrates

#### Cell seeding

Human umbilical vein endothelial cells (HUVECs) were cultivated in EGM-2 medium and 3^rd^-4^th^ passages were used. The coated substrates were sterilized by UV radiation for 1 h prior to HUVECs seeding. The cells were seeded on the glass bottom of a well in 96- or 24- well plates at concentrations of 6700 or 5300 cells per cm^2^, respectively. The medium EGM-2 was depleted from FGF and VEGF and supplemented with 1% FBS and aprotinin (10 μg ml^−1^) (Medium 1). A flat sample of an ePTFE vessel wall was fixed by CellCrown™ inserts at the bottom of a well in a 24-well glass bottom plate in such a way that the luminal (inner) wall surface was exposed to a HUVECs suspension introduced above the sample. After coating and UV sterilization of the ePTFE samples, the cells were seeded at a concentration of 10 000 cells per cm^2^ in EGM-2 depleted from FGF and VEGF and supplemented with 1% FBS (Medium 2).

#### Viability of HUVECs seeded on coated glass

Fb/Hep coatings were prepared on the glass bottom of a well in a 96-well plate and modified by incubation with solutions of FGF or VEGF at concentrations of 10 ng, 100 ng, 1 μg, and 10 μg ml^−1^ in PBS or with a co-solution of FGF (100 μg ml^−1^) and VEGF (100 μg ml^−1^) in PBS for 2 h. The cell viability was measured after 3 and 7 days by Cell Counting Kit-8 (CCK-8) according to the manufacturer's manual. The colored product developed by CCK-8 was measured at 450 nm using a microplate reader.

#### Growth of HUVECs seeded on coated glass and ePTFE samples

Glass bottom of wells in a 24-well plate and ePTFE vessel samples fixed in a 24-well plate by CellCrown™ inserts were modified by Fb/Hep coatings and then incubated with solutions of FGF and/or VEGF at concentrations of 100 ng ml^−1^ in PBS for 2 h. On days 3 and 5 after seeding on glass and 5 days after seeding on ePTFE, the cells were fixed with 4% paraformaldehyde for 30 min and blocked with a 1% BSA solution. Subsequently the cells were permeabilized with 0.1% Triton-X 100 and fluorescence stained with phalloidin (1 : 100) for F-actin and Hoechst (5 μg ml^−1^) for nuclei and observed under an Olympus IX83 confocal microscope.

### Statistical analysis

The quantitative data are presented as the mean with standard deviation (±S.D). The Student's one-tailed *t*-test was performed for statistical comparisons of cell viability results, and a value of *p* < 0.05 was considered statistically significant.

## Results and discussion

### Characterization of Fb/Hep coating

Fibrin coating was grown on the surface of a glass bottom well in a 24-well glass bottom plate or ePTFE vessel prosthesis. The procedure is based on growing a controlled fibrin mesh on a material surface *via* the catalytic effect of a fibrin(ogen)-bound thrombin and the ability of antithrombin III to inhibit thrombin in solution but not fibrin(ogen)-bound thrombin.^30,35^

**Scheme 1:**
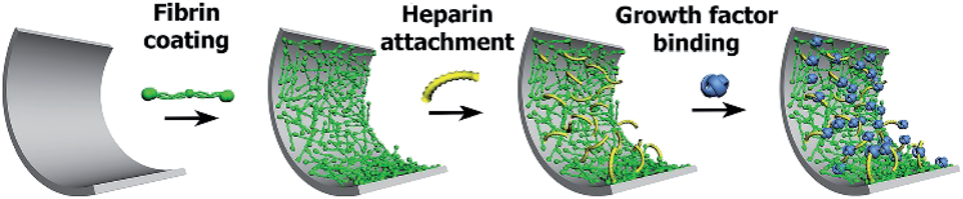
The scheme represents the preparation of Fb coating and its modification with heparin and subsequent attachment of growth factors.

**Fig. 1:**
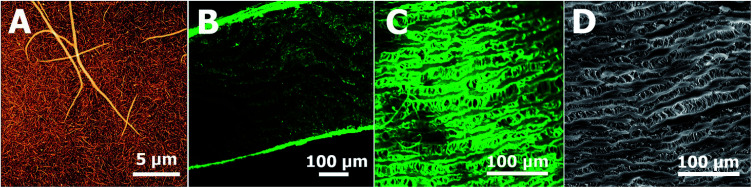
(A) Morphology of fibrin coating on glass observed using atomic force microscope. (B) Fibrin in a cross section of the coated ePTFE vessel wall (confocal microscope). (C) Fibrin on the inner surface of the coated ePTFE vessel wall (confocal microscope). (D) The inner surface of the uncoated ePTFE vessel wall (SEM). Fibrin was immunofluorescence stained with Alexa Fluor 488 (green).

The Scheme 1 illustrates successive modification of a substrate by a fibrin mesh (Fb), covalent binding of heparin (Fb/Hep) and noncovalent binding of GFs (Fb/Hep + VEGF, Fb/Hep + FGF, and Fb/Hep + FGF + VEGF). Fig. 1 shows an AFM image of a dried Fb coating on a glass surface (Fig. 1A) with a uniform fibrin mesh. The average thickness of the dried fibrin mesh measured by AFM was 40 ± 10 nm. The surface density of fibrin in the Fb coating on glass was 4.0 ± 0.7 μg cm^−2^ (evaluated by BCA assay). The activity of heparin covalently conjugated to the fibrin coating on glass (Fb/Hep) was 0.027 ± 0.02 U cm^−2^ as determined by an anti-FXa activity assay.

The confocal microscopic images of the surface and cross section of the Fb modified ePTFE vessel show both fibrin inside the vessel wall as well as fibrin homogenously decorating the surface of the ePTFE vessel (Fig. 1B and C), that copies the ePTFE morphology of inner surface on the unmodified prosthesis (Fig. 1D). SEM images in the ESI† show a homogeneous formation of the Fb localized at the surface of the ePTFE vessel prosthesis using the coating technology (Fig. S1†). The surface density of fibrin in the Fb/Hep coated ePTFE vessel was 40 ± 8 μg cm^−2^ and the heparin activity was 0.5 ± 0.1 U cm^−2^.

Several other coating techniques have been developed to cover ePTFE vascular grafts with fibrin, such as the incubation of a graft with fibrin and thrombin substances,^27^ the gelation of fibrin glue mixtures on ePTFE grafts in a vacuum,^36^ or the gelation of a fibrin–thrombin mixture under pressure in occluded grafts with the additional removal of excess fibrin on a surface.^37,38^ Unlike other techniques, our method makes it possible to reproducibly coat various types of surfaces, including the inner surface of porous materials, with fibrin meshes of thickness adjustable from nanometre to micrometre scale without the formation of a bulk fibrin gel.^30,31^

### Binding of VEGF and FGF to Fb/Hep coatings

**Fig. 2:**
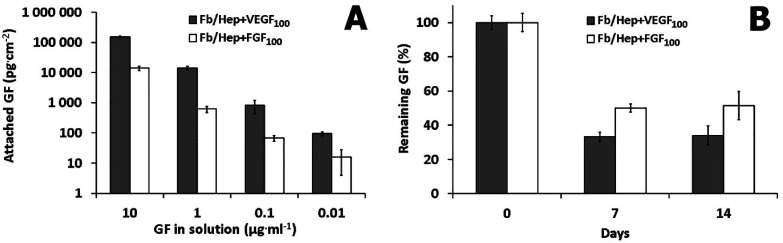
(A) Dependence of the amount of VEGF (gray columns) and FGF (white columns) attached to Fb/Hep coatings on the concentration of the loaded protein in solution. (B) Fractions of VEGF (gray columns) and FGF (white columns) remaining in Fb/Hep + VEGF_100_ and Fb/Hep + FGF_100_ coatings, after 7 and 14 days in PBS. The GFs were loaded from 100 ng ml^−1^ solutions. The error bars represent standard deviation of the mean (*n* = 3).

Amounts of VEGF or FGF attached to the Fb/Hep coatings prepared on the glass substrates are shown in Fig. 2A. The graph shows that the individual growth factors were attached to the Fb/Hep coatings in a dose-dependent manner. Furthermore, the amounts of the loaded VEGF were considerably higher than those of FGF (Fig. 2A). VEGF and FGF could bind to the Fb/Hep both *via* specific binding centers in fibrin and *via* specific interaction with immobilized heparin.^32–34^

The amounts of bound FGF and VEGF did not depend on whether these were bound individually or simultaneously. The Fb/Hep + FGF_100_ + VEGF_100_ coating contained 972 ± 160 pg cm^−2^ of VEGF and 52 ± 4 pg cm^−2^ of FGF. These values did not significantly differ from the amounts of the individually attached VEGF (815 ± 378 pg cm^−2^) and FGF (67 ± 14 pg cm^−2^) loaded from the same concentration (100 ng ml^−1^), indicating that the binding of the GFs is independent. Indeed, it has been reported that binding of VEGF to fibrin is independent of FGF.^14^

### The release of VEGF and FGF from Fb/Hep coatings

Release experiments were performed on the Fb/Hep coating loaded from 100 ng ml^−1^ solutions of VEGF or FGF. The ability of Fb/Hep coatings to retain GFs is depicted in Fig. 2B. After 7 days of incubation in PBS, 33 ± 3% of VEGF and 50 ± 3% of FGF remained bound in the Fb/Hep + VEGF_100_ and Fb/Hep + FGF_100_ coating, respectively. We did not observe any significant changes between 7 days and 14 days of incubation in PBS. After 14 days, 34 ± 6% of VEGF and 51 ± 8% of FGF remained bound in Fb/Hep + VEGF_100_ and Fb/Hep + FGF_100_, respectively. In addition, we have also measured the amount of released GFs during two weeks incubation in PBS (Fig. S2†). After a fast release during the first 24 h only a minimal release was detected for the next 14 days. The determined released amount is in line with the amount of GFs remaining on the Fb/Hep coatings after 14 days (Fig. 2B). The growth factors may be strongly immobilized by specific noncovalent binding to fibrin^14,32^ and heparin.^33,34^ In contrast to the long-term stability of covalent attachment of heparin in the Fb/Hep coatings incubated with PBS,^29^ some of the specifically bound or only nonspecifically adsorbed growth factors are only loosely bound in the coating.

### Hemocompatibility of the coatings

**Fig. 3:**
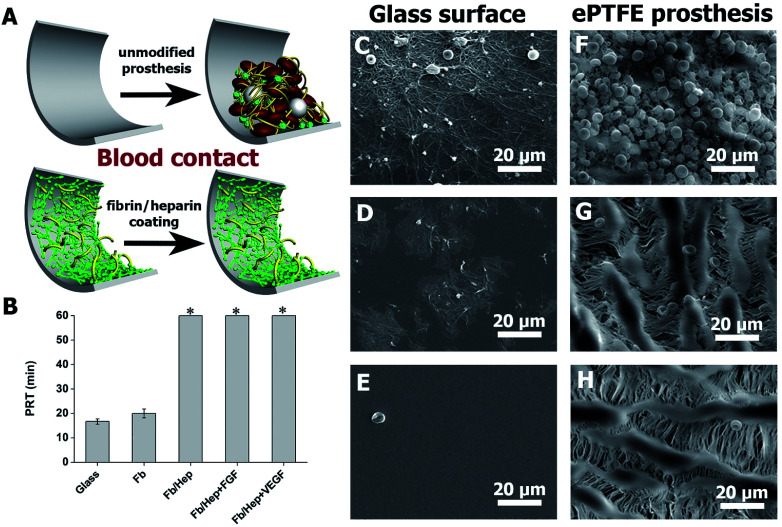
(A) Scheme showing a comparison of unmodified and fibrin/heparin modified prosthesis after blood contact. (B) Graph showing plasma recalcification times (PRT) after contact with various coatings on glass with human blood plasma recalcified with CaCl_2_ (0.02 M final concentration; mean ± S.D.; *n* = 3). * – an asterisk indicates samples where the plasma did not coagulate within 60 min. (C–E) SEM images of unmodified glass (C), Fb coated glass (D), and Fb/Hep coated glass(E), (F–H) SEM images of unmodified ePTFE vessels (F), Fb/Hep coated (G), Fb/Hep + FGF_100_ + VEGF_100_ coated (H) after 1 h incubation with fresh heparinized human blood at 37 °C.

Hemocompatibility of the coatings was tested by contact with fresh human blood and by a plasma recalcification test (PRT). The prolongation of the recalcified plasma clotting time was measured to evaluate the coating's ability to resist the activation of the clotting cascade initiated when a biomaterial comes into contact with circulating blood.^39^ The activation of coagulation of freshly recalcified plasma was indicated by the increase in plasma turbidity on the coated glass surfaces in comparison with the uncoated glass. PRT of 16 ± 1 min on the unmodified glass was prolonged by the Fb coating by up to 20 ± 2 min. The attachment of heparin in Fb/Hep did not activate coagulation within 60 min when the test was terminated. Importantly, the binding of VEGF and FGF to the Fb/Hep coatings (Fb/Hep + VEGF_100_ and Fb/Hep + FGF_100_, respectively) also did not activate the plasma coagulation within 60 min when the test was terminated (Fig. 3B). This observation clearly indicates that heparin after binding of GFs remains active and is able to suppress blood plasma activation.

Additionally, in order to mimic initial thrombogenic processes activated after the vascular prosthesis implantation, the coated glass and ePTFE vessel samples were exposed to freshly drawn heparinized human blood and the resulting blood clot was analysed by SEM. Fig. 3C–E shows representative SEM images of bare and coated glass surfaces after their exposure to heparinized blood. Fig. 3C displays a blood clot composed of platelet aggregates, leukocytes, and red blood cells entrapped in a fibrin mesh on the bare glass. Fig. 3D shows patches of spread activated platelets, some platelet aggregates and nearly no leukocytes and red blood cells on the Fb coated glass. In contrast, no blood clot deposits were formed on the Fb/Hep coated glass (Fig. 3E). The images correlate with PRT data in Fig. 3A and indicate low thrombogenicity of the Fb/Hep surface modification. Fig. 3F–H shows SEM images of the bare ePTFE and the Fb/Hep and Fb/Hep + FGF_100_ + VEGF_100_ coated ePTFE vessel wall in contact with heparinized blood.

The images display an initial thrombus with platelet aggregates and many entrapped leukocytes and red blood cells on the unmodified ePTFE vessel (Fig. 3F) and only sparsely scattered red blood cells but no platelets or leukocytes on the inner side of the ePTFE vessel coated with Fb/Hep (Fig. 3G) and Fb/Hep + FGF_100_ + VEGF_100_ (Fig. 3H). Sparsely scattered red blood cells were also observed on the ePTFE vessels modified with the Fb/Hep + FGF_100_ and Fb/Hep + VEGF_100_ coatings containing immobilized heparin (Fig. S3†).

We have previously reported on the significantly improved *in vitro* hemocompatibility of PVC substrates^29^ and neurovascular stents^40^ that had been modified with Fb/Hep coatings by a similar technique as applied here. In contact with heparinized blood in a Chandler loop or a flow loop model mimicking human blood flow, the coatings suppressed thrombin generation, platelets and leukocyte activation, complement activation and blood clot formation.^29,40^ The results presented herein indicate that the binding of GFs preserve the excellent hemocompatibility of the Fb/Hep coating.

### Viability of HUVECs on Fb coatings

**Fig. 4:**
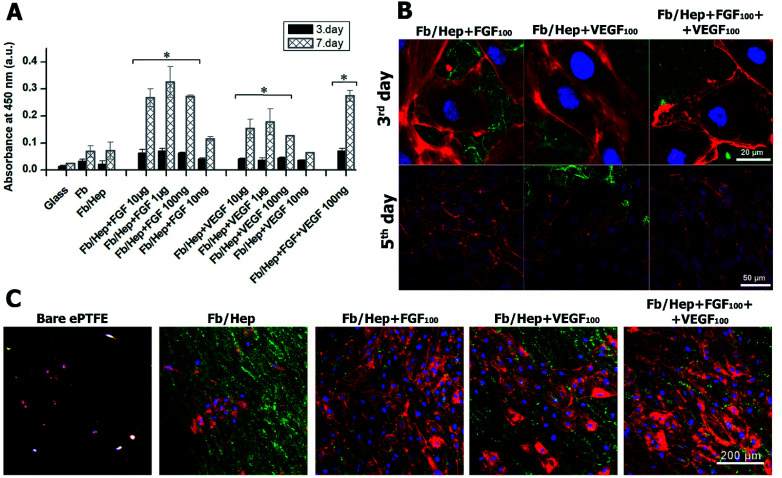
(A) The viability of HUVECs on days 3 and 7 after seeding on glass coated with Fb/Hep and modified by the attachment of growth factors from solutions containing various concentrations of FGF, VEGF, and a co-mixture of FGF (100 ng ml^−1^) and VEGF (100 ng ml^−1^). The data were obtained from four independent experiments, mean ± S.D, Student *t*-test (*p < 0.05). Representative confocal microscopy images of (B) HUVECs on day 3 and day 5 after seeding on the coated glass and (C) HUVECs on day 5 after seeding on the inner surface of the modified ePTFE vessel wall. Fb/Hep + FGF_100_, Fb/Hep + VEGF_100_, and Fb/Hep + FGF_100_ + VEGF_100_ were prepared by binding the GFs from solutions containing 100 ng ml^−1^ of the respective GF. The cells were stained with phalloidin for F-actin filaments (red) and with Hoechst for nuclei (blue); fibrin was immunofluorescence stained with Alexa Fluor 488 (green).

The viability of HUVECs on days 3 and 7 after the seeding on the coated glass was characterized by measuring the cell metabolic activity. The results are presented in Fig. 4. The glass bottoms of wells were coated with Fb/Hep and loaded with FGF or/and VEGF at various concentrations (10 ng ml^−1^ to 10 μg ml^−1^, see Methods). Both Fb and Fb/Hep coating promoted the HUVEC viability compared to bare glass. This is in line with previously reported data that surface-attached fibrin improved adhesion and differentiation of endothelial cells.^41^ Moreover, a similar increase in HUVECs viability was obtained for the Fb or Fb/Hep coating thus indicating that bound heparin did not interfere with cell proliferation. A significant increase in the cell viability was reached if Fb/Hep coatings were loaded with FGF or VEGF from solutions containing the GFs at concentrations of 10 μg, 1 μg, or 100 ng per 1 ml (Fig. 4A). Relatively small differences in the cell viability on coatings loaded from these solutions contrasted with the huge differences in the initial amounts of VEGF or FGF bound in the respective coatings (Fig. 2A).

Therefore, the Fb/Hep coatings loaded with the GFs from 100 ng ml^−1^ solutions (referred as Fb/Hep + VEGF_100_; Fb/Hep + FGF_100_; and Fb/Hep + VEGF_100_ + FGF_100_) were selected for further cell experiments because of their blood compatibility (Fig. 3) and the sufficient viability of HUVECs seeded on their surfaces (Fig. 4A). The release of GFs from the coatings (Fig. 2B, S2†) indicates that a considerable increase in the cell viability between day 3 and 7 may be due to the activity of a fraction of the GFs remaining immobilized in the coatings after the initial fast release. Remarkably, FGF promoted almost twice as much cell viability than VEGF, even if the initial FGF amounts in the coatings were considerably lower (Fig. 2A), *e.g.* Fb/Hep + FGF_100_ contained 67 pg cm^−2^ of FGF, while Fb/Hep + VEGF_100_ contained 815 pg cm^−2^ of VEGF. In accordance with our previous report, we did not observe any significant differences in cell viability between the Fb and Fb/Hep coating, indicating that the immobilized heparin did not have any undesirable effect on cell growth.^25^

### Morphology of HUVECs on fibrin coatings

#### Fibrin coating on glass

The Fb/Hep coatings loaded with VEGF and FGF on glass or ePTFE prosthesis were seeded with HUVECs and observed after 3 and 5 days by a confocal microscope. Fig. 4B shows representative confocal microscopy images of HUVECs seeded on the coated glass samples that were immunofluorescently stained for cell actin (red), cell nuclei (blue) and the fibrin (green). Areas coated with fibrin (green) among HUVECs colonies were visible on day 3 on all tested surfaces, *i.e.* Fb, Fb/Hep, Fb/Hep + VEGF_100_, Fb/Hep + FGF_100_ and Fb/Hep + FGF_100_ + VEGF_100_. Moreover, the F-actin filaments (red) associated with external cell membranes demonstrated the typical cobblestone structure of the HUVECs in the cell colonies.^42^ On day 5, confluent layers of HUVECs were formed on Fb/Hep + FGF_100_ and Fb/Hep + VEGF_100_ + FGF_100_. Cells in the layers were densely packed and had elongated shapes due to competition between the rapidly proliferating cells for an available surface. VEGF and FGF has a synergistic effect on angiogenesis,^43–45^ however; we did not observe significant differences in the viability and morphology of HUVECs seeded on Fb/Hep + FGF_100_ and on Fb/Hep + FGF_100_ + VEGF_100_ on a glass substrate. We assume that this could be a consequence of a higher FGF stimulating potency compared to VEGF on HUVECs proliferation. In addition, the VEGF retained on the coating, after endothelial cells attachment, can potentially stimulate the abluminal VEGFRs receptors of ECs to induce permeability instead of supporting proliferation.^46^ In contrast the HUVEC cells on Fb/Hep + VEGF_100_ coating were more spread than cells on Fb/Hep + FGF_100_ (Fig. S4†). The effect of FGF and VEGF loaded to the Fb/Hep coating at various concentrations on cell proliferation and morphology is shown in ESI Fig. S5† and the effect of both GFs agreed with the cell viability experiments (Fig. 4A). The cell reached a confluent layer after 5 days at a concentration of 100 ng ml^−1^. Further increase in the amount of VEGF had only a mild effect on cell proliferation, and higher concentrations of FGF led to densely packed cells with elongated shapes. There was a clear correlation between cell proliferation and high cell viability on the FGF containing coatings and lower viability on Fb/Hep + VEGF_100_ (Fig. 4A, S4 and S5†).

#### Fibrin coatings on ePTFE

**Fig. 5:**
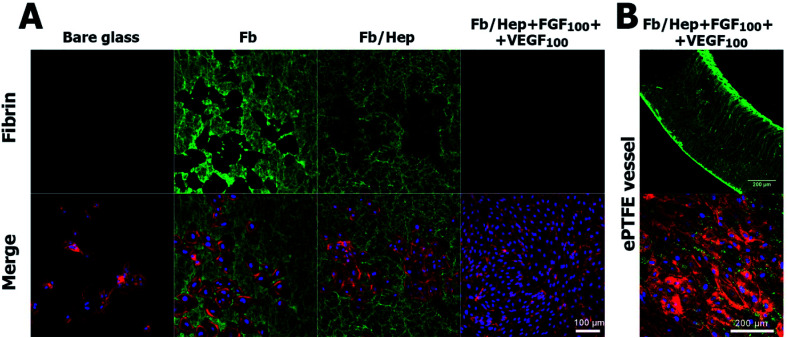
Degradation of fibrin coating observed by confocal microscopy on day 5 after seeding HUVECs on coated glass (A) and on an ePTFE vessel (B). Upper row: (A) fibrin visualized on bare glass and glass coated with Fb, Fb/Hep, Fb/Hep + FGF_100_ + VEGF_100_ prepared by the attachment of the GFs from a solution containing 100 ng ml^−1^ FGF and 100 ng ml^−1^ VEGF. (B) Fibrin visualized in a cross section of an ePTFE vessel wall modified by Fb/Hep + FGF_100_ + VEGF_100_. Lower row: (A) merged image of fibrin and HUVECs visualized on bare and coated glass; (B) and on the inner surface of the ePTFE vessel wall. Fibrin was immunolabelled with Alexa Fluor 488 (green), and HUVECs were stained with phalloidin for actin (red) and with Hoechst for nuclei (blue).

Fig. 4C shows confocal microscopy images of HUVECs on day 5 after seeding the HUVEC on the inner surface of the ePTFE vessel wall. There was only a small amount of non-spread cells on the surface of the bare ePTFE, a few HUVECs colonies on Fb/Hep, large colonies on Fb/Hep + VEGF_100_, and nearly confluent HUVECs layers on Fb/Hep + FGF_100_ and Fb/Hep + FGF_100_ + VEGF_100_. No cells were found inside the vessel wall (Fig. 5) observed in cross section, probably due to the incapability of the cells to penetrate through the ePTFE wall. It is important to mention that the cells were cultivated in a medium depleted from growth factors. This condition is important to represent the potential of the coating for implantation, as no systemic GF delivery is feasible in bloodstream. Therefore, only localized release of GFs from the coating can be applied to enhance endothelialization.

VEGF and FGF have been widely studied due to the essential role of growth factors in wound healing, angiogenesis and tissue engineering.^43–45^ The importance of FGF for HUVECs viability on the coated glass (Fig. 4A) and HUVECs growth on the ePTFE vessels (Fig. 4C) may correlate with the higher potency of FGF compared to VEGF to stimulate microvascular endothelial cells and HUVECs to form endothelial vessel-like tubes in a collagen matrix.^44^ The capacity of FGF to stimulate ECs proliferation in our experiments was probably further enhanced by the FGF attachment to fibrin.^45^ Contrary to the potent synergism between VEGF and FGF in the induction of the angiogenesis both *in vitro*,^43,44^ and *in vivo*,^45^ we did not find significant differences in the viability and growth of HUVECs seeded on Fb/Hep + FGF_100_ and on Fb/Hep + FGF_100_ + VEGF_100_. The VEGF effect on angiogenesis is associated with increased vascular permeability in endothelial cells.^46^ On the other hand, the presence of bound VEGF in Fb/Hep + FGF_100_ + VEGF_100_ coating may be important for capturing EPCs from peripheral blood, their homing and further differentiation into ECs.^17,47^ Recently it has been reported that immobilized VEGF also captures blood circulating monocytes and initiates their differentiation into a mixed endothelial (EC) and macrophage phenotype that further develops into mature EC *in vivo*.^48^

Moreover, ECs pre-seeded on fibrin-coated ePTFE grafts exhibited better resistance to shear stress than the control.^38,49^ Similarly, the coating of PET vascular grafts with fibrin using the technique described herein significantly increased the resistance of ECs seeded on the grafts to shear stress.^50^

#### Degradation of the fibrin coating

The gradual degradation of fibrin was observed by confocal microscopy below HUVEC colonies on all fibrin coatings 5 days after seeding the cells (Fig. 5A and S4–S6†). Fibrin areas (green) were still visible among HUVEC colonies and no fibrin was observed below the HUVEC colonies on the Fb and Fb/Hep coatings (Fig. 5A). The surface completely free of fibrin (black) was observed below cell monolayer on Fb/Hep + FGF_100_ and Fb/Hep + FGF_100_ + VEGF_100_ (Fig. 5A, S4 and S5†). The absence of fibrin below the cells is depicted by complementary patterns of fibrin and cells (Fig. 5A).

Although the complete degradation of the Fb/Hep + FGF_100_ + VEGF_100_ coating below HUVECs was revealed on glass, some fibrin was still visible inside and on the surface of the ePTFE vessel prosthesis 5 days after seeding with HUVEC. The image of a cross section of the ePTFE vessel wall modified by Fb/Hep + FGF_100_ + VEGF_100_ (Fig. 5B, upper row) shows fibrin remaining inside the vessel wall and in a denser structure at the wall surface. An almost confluent layer of HUVECs with traces of fibrin was visible on the inner ePTFE vessel wall surface (Fig. 5B, lower row) and no cells were observed inside the wall. Evidently, HUVECs could not penetrate through a dense structure formed by PTFE nodes and fibrils at the inner wall surface (Fig. 1D).

The findings indicated that fibrinolysis occurred mainly in a close vicinity of the proliferating HUVECs. Such a fibrinolytic system contributes to the degradation of blood clots formed to stop bleeding after injury. During the process fibrin is lysed by serine protease plasmin. Plasmin is produced by the conversion of inactive zymogen plasminogen from circulating blood. The conversion is performed by tissue plasminogen activator (tPA) attachment of plasminogen and tPA to fibrin on the cell surfaces.^51,52^ In our *in vitro* experiments, fibrin in contact with proliferating HUVECs could have been cleaved by plasmin produced by the conversion of plasminogen from 1% serum contained in the culture medium. Alternatively, fibrin may be degraded by extracellular zinc-dependent endopeptidases responsible for the remodelling of the extracellular matrix, including fibrin (matrix metalloproteinases) secreted by HUVECs or that present in the serum.^53–55^ Additionally, the fibrinolysis may be potentially activated by VEGF stimulating the abluminal VEGFRs receptor of ECs.^54^ Regrettably, our experiments did not allow us to evaluate the decrease in the amount of fibrin under HUVECs growing on the inner surface of the ePTFE vessel wall as opposed to that on the coated glass. Nevertheless, we expect that a gradual degradation of the fibrin remaining inside the vessel wall might occur by a slow diffusion of fibrinolytic proteases from circulating blood and expressed by the cells growing on the ePTFE wall surface.

Therefore, covalently bound heparin and strongly noncovalently attached growth factors may be released from the coating remaining in the ePTFE vessel and prolong the capacity of the vessel to support endothelialization and suppress the thrombus formation after the implantation.^14,56^

## Conclusions

In this study, we presented a new technique for surface modification of vascular prostheses that is based on the controlled growing of a fibrin mesh from fibrinogen solutions catalysed by thrombin immobilized on a substrate and the subsequent covalent binding of heparin and noncovalent binding of growth factors FGF and VEGF. This approach allows to coat reproducibly the surface and interior of a standard ePTFE vessel prosthesis with a controlled fibrin coating without the formation of a bulk fibrin gel. The coating completely prevents blood clot formation on the inner surface of the prosthesis exposed to heparinized fresh human blood *in vitro* and accelerates the creation of a monolayer of HUVECs seeded on the surface. Viability of the cells is promoted mainly by FGF immobilized in the Fb/Hep + FGF and Fb/Hep + FGF + VEGF coatings. The cells do not penetrate the ePTFE vessel wall and can degrade the coating of the wall surface by fibrinolytic processes localized at their surfaces. The coating remaining inside the vessel wall has probably been only slowly degraded by fibrinolytic agents diffusing from the wall surface. The obtained findings suggest that the developed coating, which is generally applicable to the modifications of various cardiovascular grafts, may prevent acute thrombus formation and support self-endothelialization of implanted ePTFE vessel prostheses and other grafts *in situ*. Additionally, the release of heparin and growth factors from the vessel wall may prevent thrombosis after the implantation of prostheses pre-seeded *in vitro* with autologous ECs and may support the transformation of the *in vitro* formed ECs layer to a functional endothelium.

## Author contributions

JT prepared the samples, performed cell experiments and wrote the manuscript. ZR designed and wrote the manuscript. EB wrote the manuscript. PM performed the hemocompatibility tests. TR performed the AFM and SEM measurements, performed the hemocompatibility tests, and designed and wrote the manuscript.

## Conflicts of interest

There are no conflicts to declare.

## Supplementary Material

RA-011-D1RA00053E-s001
