# Supplementary material for: Endothelialization of an ePTFE vessel prosthesis modified with an antithrombogenic fibrin/heparin coating enriched with bound growth factors
Source: RSC Adv. 2021 Feb 3;11(11):5903–13. doi: 10.1039/d1ra00053e (PMC8694727; doi:10.1039/d1ra00053e)
Supplement: RA-011-D1RA00053E-s001 [file RA-011-D1RA00053E-s001.pdf]

Electronic Supplementary Material (ESI) for RSC Advances.
This journal is © The Royal Society of Chemistry 2021

Electronic Supplementary Information

Endothelialisation of ePTFE vessel prosthesis modified with an antithrombogenic fibrin/heparin coating enriched with bound growth factors

J. Táborská[1], Z. Riedelová[1], E. Brynda[1], P. Májek[2], T. Riedel[1]*

[1]Institute of Macromolecular Chemistry, Czech Academy of Sciences

[2]Institute of Haematology and Blood Transfusion*

Corresponding author: riedel@imc.cas.cz

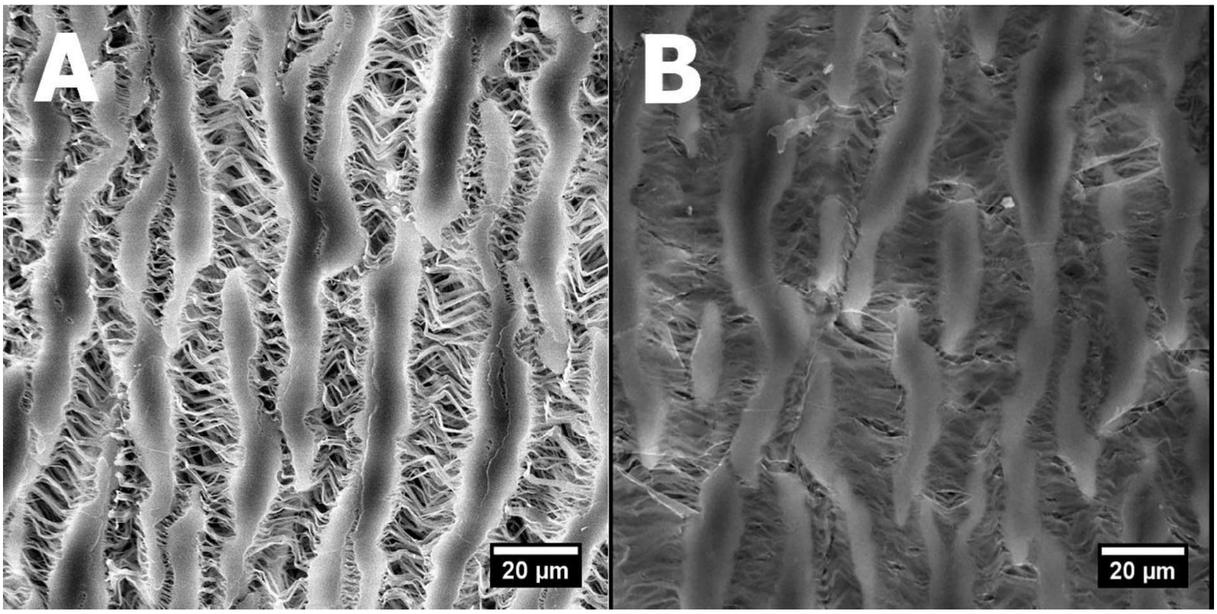

Figure S1. Fb coatings on ePTFE vessel prosthesis. A) inner surface of unmodified ePTFE prosthesis; B) inner surface of Fb coated prosthesis obtained using scanning election microscopy (SEM).

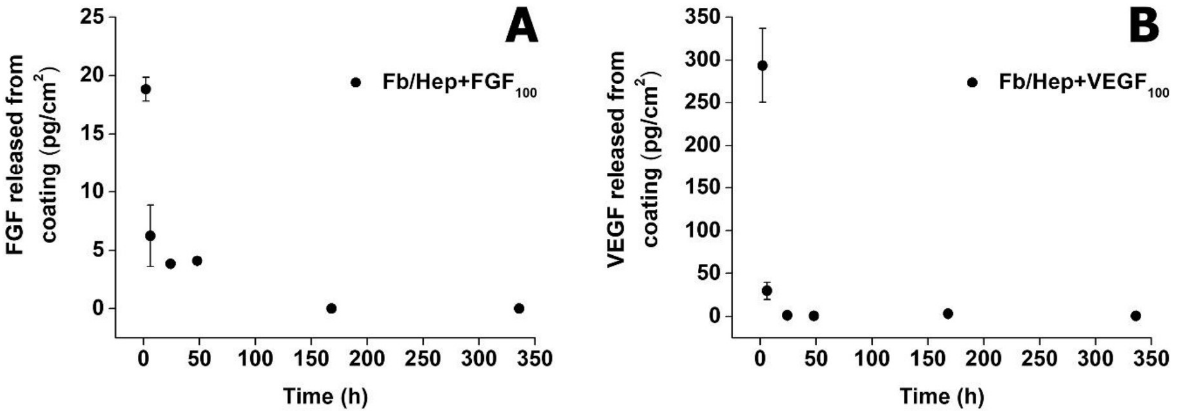

Figure S2. Release of growth factors from Fb/Hep coatings into PBS. The coatings were prepared on glass from solutions containing 100 ng/ml of VEGF or FGF. The error bars represent standard deviation of the mean (n=3).

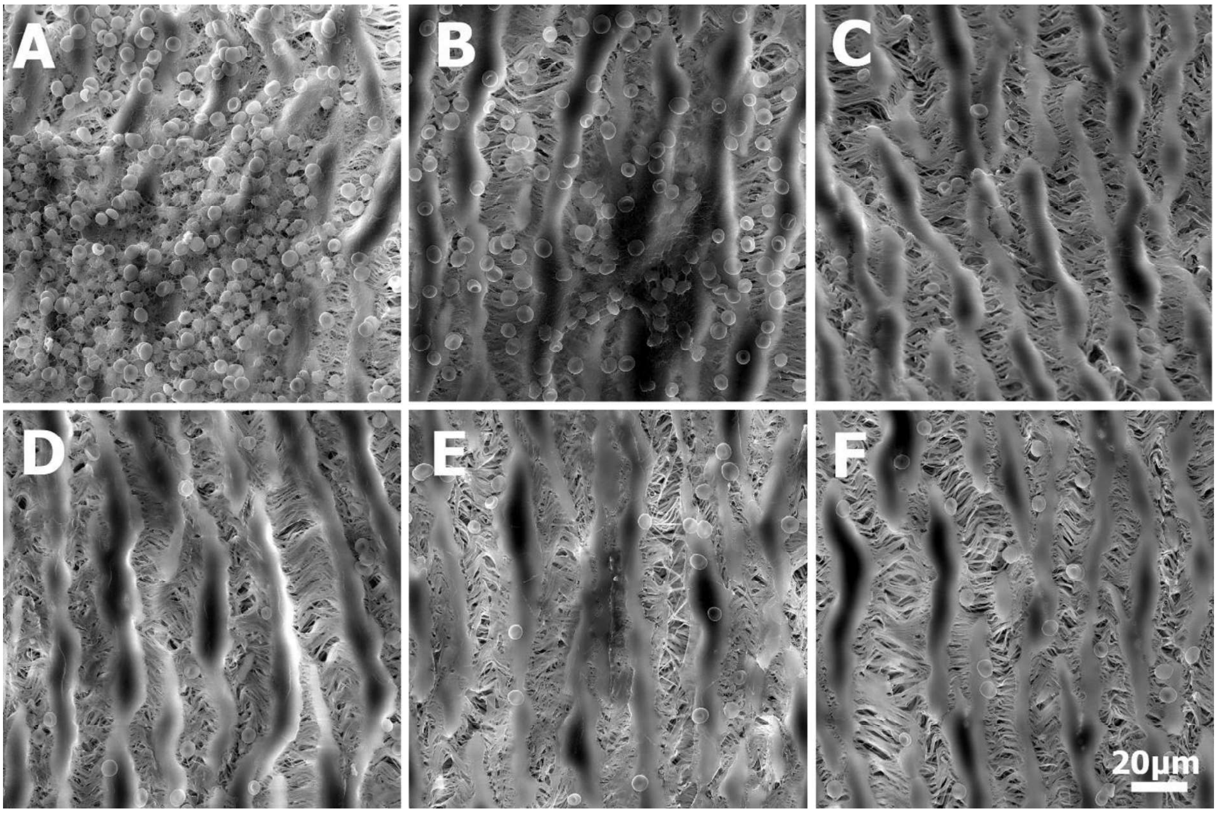

Figure S3. Inner surface of the unmodified and fibrin modified ePTFE vessel after 1 h contact with fresh heparinized (1 U/ml) human blood at 37°C. SEM images of A) unmodified ePTFE; B) Fb modified ePTFE; C) Fb/Hep modified ePTFE; D) Fb/Hep+FGF100 modified ePTFE; E) Fb/Hep+$VEGF_{100}$ modified ePTFE; and F) Fb/Hep+FGF_{100}+$VEGF_{100}$ modified ePTFE. SEM of dried samples.

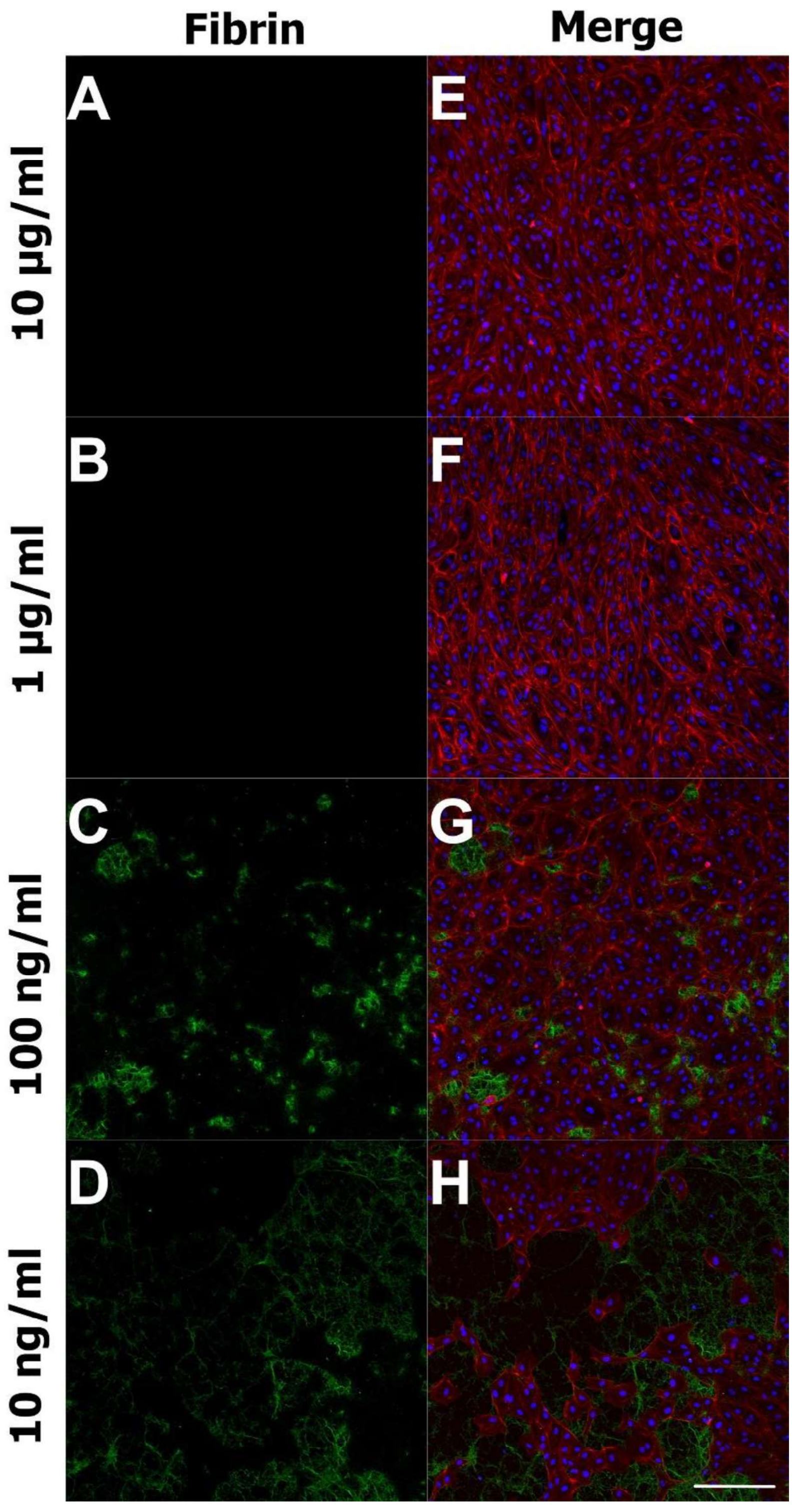

Figure S4. HUVEC on day 5 after seeding on the bare glass coated with the Fb/Hep coating containing increasing concentration of VEGF. The cells were stained with phalloidin for F-actin filaments (red) and with Hoechst for nuclei (blue); fibrin remaining on glass was immunofluorescence stained with Alexa 488 (green); confocal microscope scale bar 200 µm.

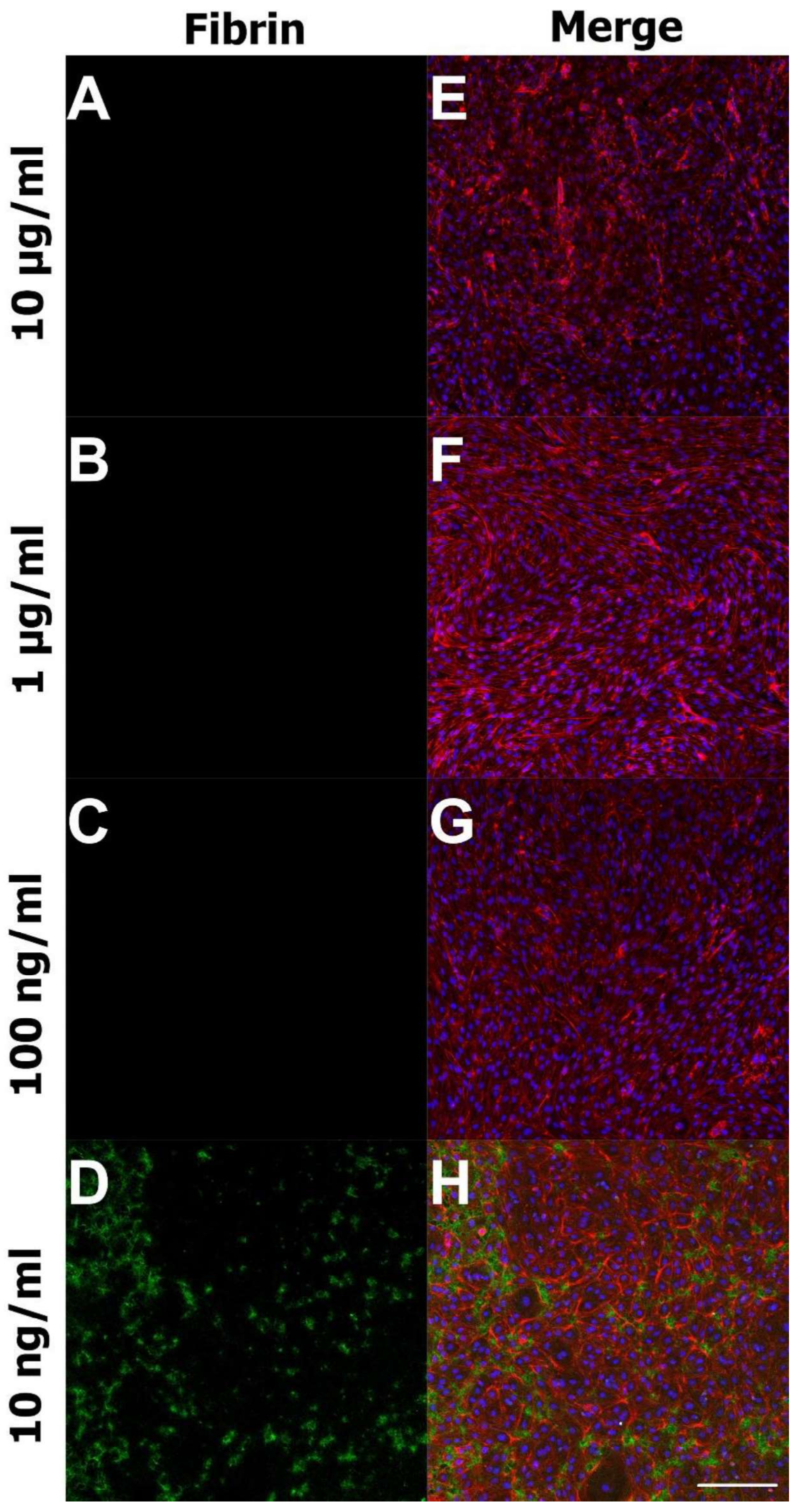

Figure S5. HUVEC on day 5 after seeding on bare glass coated with the Fb/Hep coating containing increasing concentration of FGF. The cells were stained with phalloidin for F-actin filaments (red) and with Hoechst for nuclei (blue); fibrin remaining on the glass was immunofluorescence stained with Alexa 488 (green); confocal microscope scale bar 200 µm.

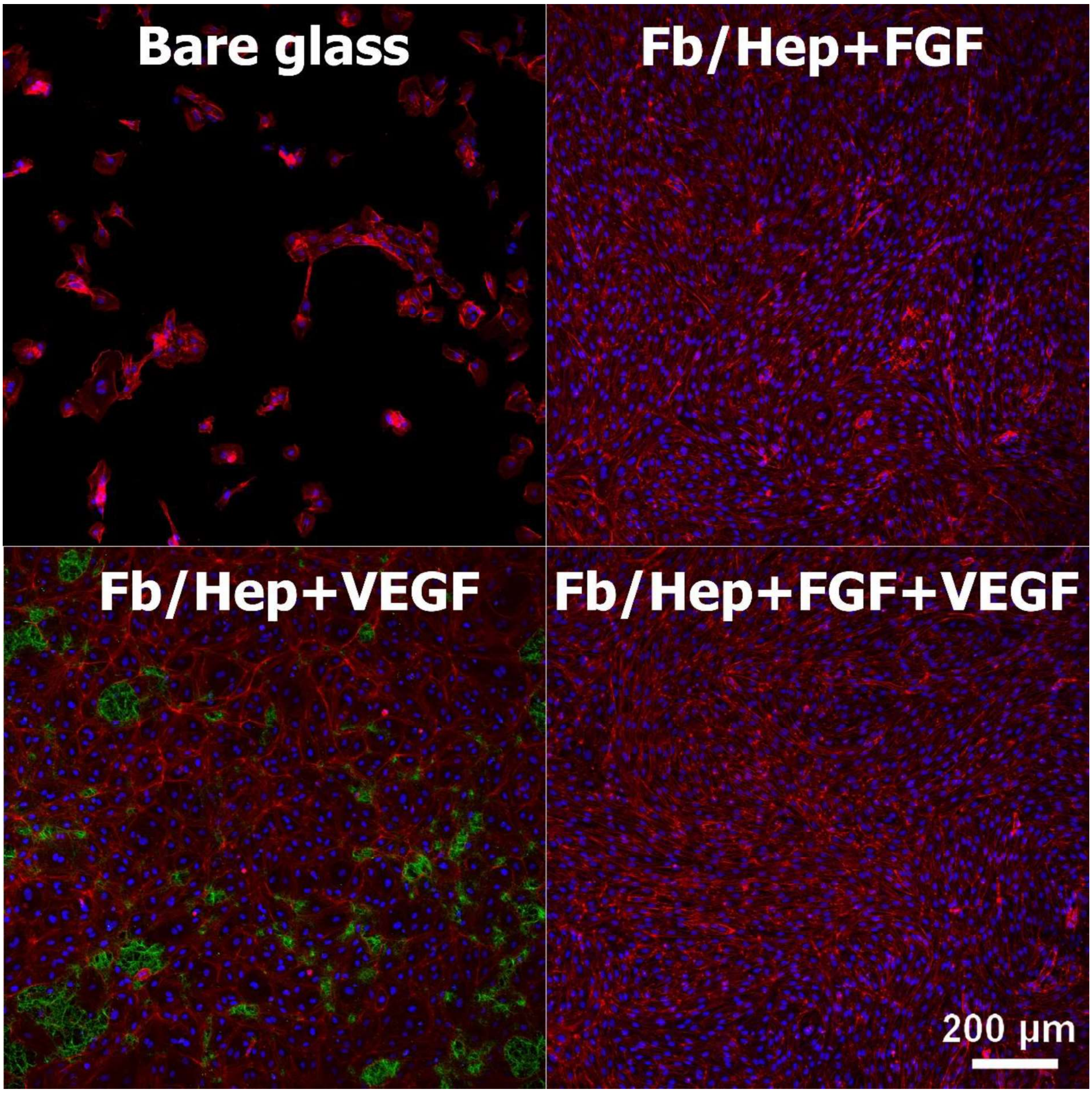

Figure S6. HUVEC on day 5 after seeding on bare glass; Fb/Hep+FGF; Fb/Hep+VEGF; Fb/Hep+FGF+VEGF. Both FGF and VEGF were deposited from a PBS solution at a concentration 100 ng/ml. The cells were stained with phalloidin for F-actin filaments (red) and with Hoechst for nuclei (blue); fibrin remaining on glass was immunofluorescence stained with Alexa 488 (green); confocal microscope scale bar 200 µm.
